# Accurately Locating Metastatic Foci in Lymph Nodes With Lugol’s Iodine-Enhanced Micro-CT Imaging

**DOI:** 10.3389/fonc.2021.594915

**Published:** 2021-09-16

**Authors:** Cheng-Wan Xia, Shi-Qi Hu, Qun-Zhi Zhou, Rong-Lin Gan, Jiong-Ru Pan, Qian Zhang, Yu-Mei Pu, Shen Chen, Qin-Gang Hu, Yu-Xin Wang

**Affiliations:** ^1^Department of Oral and Maxillofacial Surgery, Nanjing Stomatological Hospital, Medical School of Nanjing University, Nanjing, China; ^2^Department of Stomatology, The Suzhou Hospital affiliated to the Nanjing Medical University, Suzhou, China; ^3^Department of Oral Pathology, Nanjing Stomatological Hospital, Medical School of Nanjing University, Nanjing, China

**Keywords:** Lugol’s iodine, micro-CT imaging, 3-dimensional imaging, metastatic lymph nodes, oral squamous cell carcinoma

## Abstract

**Background:**

Accurate evaluation of lymph node (LN) status is the key factor to determine the treatment and evaluate prognosis for patients with cancer. However, traditional pathological examination resulted in a 30% false-negative rate of detection of metastases in LNs. This study aimed to utilize Lugol’s iodine (I_2_-IK)-enhanced micro-CT imaging to reveal the 3-dimensional structure of regional LNs and decrease the false-negative rate in pathological examination.

**Methods:**

To explore the feasibility of I_2_-IK-enhanced micro-CT imaging in locating metastatic lesion in LNs, nonmetastatic and metastatic LNs from mice were used to mimic the imaging process. Then, the LNs from oral squamous cell carcinoma (OSCC) patients were applied to verify the value of I_2_-IK-enhanced micro-CT imaging in revealing LN structure and locating metastatic lesions in LNs. The glycogen content in nonmetastatic and metastatic LNs was further detected by the use of a glycogen assay kit and periodic acid–Schiff (PAS) staining to explain the imaging differences between them.

**Results:**

In nude mice, 0.5% I_2_-IK staining for 4 h was the best parameter for normal LN. The metastatic foci in metastatic LNs were also clearly outlined in this condition. For nonmetastatic LNs from patients with OSCC, 1% I_2_-IK staining for 12 h was the best parameter. However, due to the increased volume of metastatic LNs, the image effect of 3% I_2_-IK staining for 12 h was superior to 1% I_2_-IK staining [tumor background ratio (TBR), 3% *vs*. 1%, 1.89 ± 0.10 *vs*. 1.27 ± 0.07, p < 0.001]. Compared with subsequent pathological sections, we found the CT intensity of metastatic foci in LNs and muscle tissues was significantly higher than in nonmetastatic regions. Meanwhile, the glycogen content of metastatic foci in LNs detected was also significantly higher than in nonmetastatic region.

**Conclusions:**

I_2_-IK-enhanced micro-CT imaging could identify the spatial location of metastatic foci in LNs. This will be an effective method to assist in decreasing the LN false-negative rate for cancer pathology.

## Introduction

Metastasis is closely related to the prognosis of patients with malignant cancers (1). Among them, regional lymph node (LN) metastases are more common than distant metastases in many kinds of cancer, such as oral squamous cell carcinoma (OSCC), gastric carcinoma (GC), and melanoma (2–4). According to previous literature, the 5-year survival rate of patient with N_+_ (the presence of metastatic LNs) was 10–30% lower than in patient with N_0_ (the absence of metastatic LNs) (1). Meanwhile, the treatment regimens also vary widely depending on the status of regional LNs. Take OSCC as an example; tumors in T1–T2 stages without LNs metastases are often treated with elective neck dissection, while radical neck dissection and postoperative radiotherapy are recommended for tumors with LNs metastases (5, 6). Hence, it is important for treatment plan formulation and prognosis estimation to accurately assess the status of regional LNs.

Imageological examination including CT, MRI, PET/CT, and ultrasound were the routine methods used to determine the status of regional LNs preoperatively (7–10). However, limited by the spatial resolution, small metastatic foci in lymph nodes tended to be missed. For example, the false negative rate of metastatic LNs was up to 20–30% in early tongue cancer (stage I/II) using imageological examination (5). At present, pathological examination is the standard used to evaluate the status of LNs. Subjected to the slicing process, micrometastasis lesions in LNs are often missed by routine sections. Serial sections of whole LNs are considered to be the best way to reduce the possibility of omissions, but it is unrealistic due to the high cost and time consumption in clinical practice (11). Sentinel LNs biopsy (SLNB) offers the benefit of identifying nodes at greatest risk for metastatic spread, decreasing morbidity caused by localized nodal dissection and has become the standard of care in breast cancer and melanoma (12, 13). However, the application value of SLNB still relied on a reliable method to detect the micrometastasis (14). More accurate approaches should be explored to avoid false negative findings.

With the development of technology, the spatial resolution of micro-CT reached the micron scale. It brings the possibility of detecting minute tumor lesions. In the study of scanning breast cancer tissue samples, Tang et al. found that micro-CT could improve the detection number of axillary LNs (15). Moreover, micro-CT imaging also plays an important role in determining the surgical margin status of breast/lung cancer intraoperative (16–18). However, due to the similar tissue density between normal tissue and metastatic tissue in LNs, it is difficult to evaluate the status of LNs and determine the spatial location of metastatic foci with current imaging technology. Due to the low price, Lugol’s iodine (I_2_-IK) has already been widely used to assist micro-CT to delineate the internal fine anatomic structure of various soft tissue samples (19–23). In oncology, I_2_-IK-enhanced micro-CT imaging has also been used to provide the 3D structural information in adamantinomatous craniopharyngioma (24) and OSCC (25). However, no study has reported the use of I_2_-IK enhanced micro-CT imaging to accurately locate the metastatic foci.

In this study, we aimed to explore the value of I_2_-IK-enhanced micro-CT in evaluating the status of regional LNs in OSCC. First, nonmetastatic LNs from nude mice and metastatic LNs from a murine melanoma model underwent micro-CT imaging stained with 0%–2% I_2_-IK for 0.5–24 h. The result showed that I_2_-IK-enhanced micro-CT imaging could outline the spatial position of metastatic foci in LNs. After that, nonmetastatic and metastatic LNs from patients with OSCC were acquired to further evaluate the feasibility in the clinic. In the end, the content of glycogen in metastatic and nonmetastatic LNs was also detected to preliminarily illustrate the proper mechanism of I_2_-IK-enhanced micro-CT imaging.

## Materials and Methods

### Nonmetastatic and Metastatic LNs From Mice

Balb/c nude mice (male, 25 g) were purchased from the Comparative Medical Center of Yang Zhou University. Melanoma cell line B16 was a gift from the College of Engineering and Applied Sciences, Nanjing University. B16 cells were cultured in Dulbecco’s modified Eagle’s medium (DMEM)/high glucose media supplemented with 10% (v/v) bovine serum, 1% (v/v) penicillin, and 1% (v/v) streptomycin. Cells were incubated in a humidified incubator at 37°C with 5% CO_2_. For the melanoma LNs metastasis model, 1 × 10^6^ B16 cells were injected into the footpad region of the hind limb of 6–7-week-old male Balb/c nude mice. Three weeks later, ipsilateral popliteal metastatic LNs were dissected for further study. For nonmetastatic LNs, 20 popliteal LNs from 10 Balb/c nude mice were dissected. All animal procedures were performed in accordance with the Guidelines for Care and Use of Laboratory Animals of Nanjing University and approved by the Animal Ethics Committee of Nanjing.

### Nonmetastatic and Suspected Metastatic LNs From Clinical OSCC Patients

This study was approved by the Medical Ethics Committee of the Institute Affiliated Stomatology Hospital, Nanjing University Medical School. Eight nonmetastatic and eight metastatic LNs were acquired from three cN0 patients and one cN+ patients with OSCC. All four patients were informed and signed the informed consent form. After fixation with formalin 12 h, all LNs were first sampled half by the pathology department to ensure pathological diagnosis, and the residual half were acquired for further study.

### I_2_-IK Staining

I_2_-IK (w/v, the concentration of iodine was 15%) was purchased from Shandong Lvying Chemical Technology Co. Ltd, China. For mice LNs staining, various concentrations of I_2_-IK including 0%, 0.5%, 1%, and 2% (w/v, the concentration of iodine) were prepared by diluting Lugol’s iodine mother liquor with 4% formalin solution. For clinical LNs staining, 1% and 3% (w/v, the concentration of iodine) Lugol’s iodine were prepared. All Lugol’s iodine solutions were preserved at room temperature and maintained in the dark.

For staining of nonmetastatic LNs from mice and patients, all LNs were divided into different groups randomly and stained for 24 and 36 h, respectively. During the stain period, LNs from mice were scanned with micro-CT at 0.5, 1, 2, 4, 8, and 24 h, while LNs from patients were scanned with micro-CT at 0, 12, 24, and 36 h. After the optimal I_2_-IK staining parameters were determined, metastatic LNs from mice were stained with 0.5% I_2_-IK for 4 h, while those from patients were stained with 1% and 3% I_2_-IK for 12 h. After the staining, all LNs were scanned with micro-CT.

### Micro-CT Scan and Quality Assessment of CT Image

A small animal micro-CT (Hiscan XM, Suzhou Heisfeld Information Technology Co. Ltd., China) was used for all CT imaging. Micro-CT scan parameters were as follows: power, 8 W; voltage, 60 V; electric current, 133.3 μA; detector mode, binging, 2 × 2; slice thickness, 50 μm; and repetition time, 75 ms. All CT image data were processed by SeProcessPro Version.1 software (Version 1.0, Suzhou Heisfeld Information Technology Co. Ltd., China). For imaging quality of nonmetastatic LNs, CT value in the periphery and in the center of LNs and CNR (contrast-to-noise ratio, CNR = CT_periphery_/CT _center_) was adopted (26).

### H&E Staining and pan-CK Immunohistochemical Staining

After I_2_-IK-enhanced micro-CT imaging, all LNs were embedded in paraffin using the standard method. Then, a series of 4-μm sections were prepared according to CT image data. For H&E staining, all sections were stained by an automatic, pathological section staining machine. For pan-CK immunohistochemical staining, paraffin sections were deparaffinized and dehydrated using a series of graded ethanol. For antigen retrieval, the sections were heated in a microwave oven with a 10 mM citrate buffer solution (pH = 6) for 10 min. Endogenous peroxidase activity was quenched by incubating the sections in 0.3% H_2_O_2_ for 5 min. After being blocked with 3% bovine serum albumin for 1 h at room temperature, the sections were incubated with anti-pan-CK (1:400, Cat. no. ab80826, Abcam) primary antibodies at 4° overnight and gently washed three times in 1× phosphate-buffered saline (PBS). Sample sections were then incubated with goat-antimouse second antibody (1:10,000, Cat. no. ab205719, Abcam) for 2 h at room temperature. The signal was developed using the horseradish peroxidase (HRP) substrate 3,3′-diaminobenzidine (DAB). Nuclear counterstain was done using hematoxylin.

### Glycogen Content

The glycogen content of normal LN tissue and metastatic tumor tissue was detected with the Liver/Muscle Glycogen assay kit (Nanjing Jiancheng Bioengineering Institute, China) according to the product manual and periodic acid–Schiff (PAS) staining. For PAS staining, a series of frozen sections were prepared from the fresh tissue and fixed with ice-cold acetone for 5 min. After natural air drying, the sections were treated with potassium periodate solution for 10 min and rinsed with water for 5 min. Then, Schiff solution was added and stained for 15 min with water rinsed for 10 min. Nuclear counterstain was done using hematoxylin.

### Statistical Analysis

Statistical analysis was performed using SPSS statistical software (version 23.0, IBM, Chicago, IL, USA). Values were presented in mean ± standard derivation (SD). Multivariate repeated measures ANOVA was used to compare the value of CT in the periphery and center region of nonmetastatic LNs and CNR. Paired t-test was used to compare the value of CT in normal and tumor tissues in the mice metastatic LNs. Student’s t-test was used to compare the value of CNR in the 3% and 1% groups in the clinical metastatic LNs. p < 0.05 was considered significant.

## Results

### 0.5% I_2_-IK Staining for 4 h Was the Optimal Parameter for Micro-CT Imaging of Normal LNs From Mice

**Figure 1:**
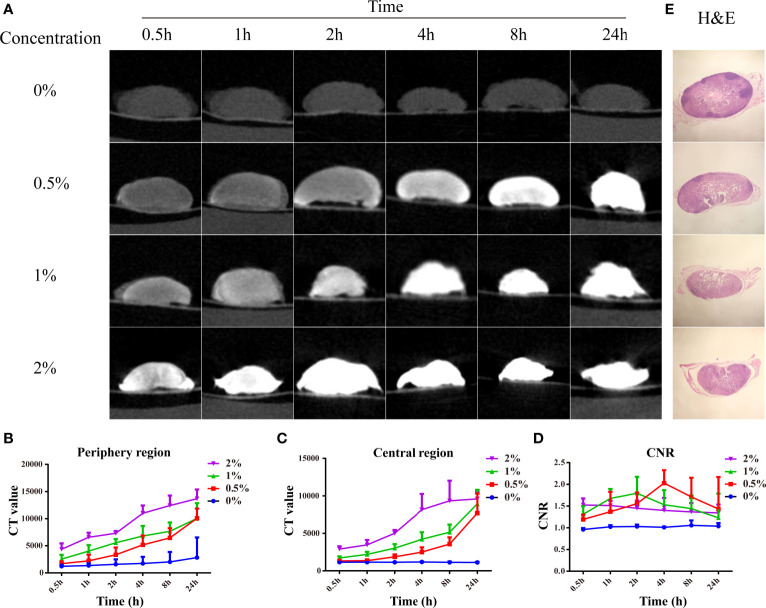
I_2_-IK-enhanced micro-CT imaging for normal LNs from mice. **(A)**, Micro-CT image of normal LNs stained with different concentration of I_2_-IK for different time. **(B, C)**, CT value in the periphery region and center region of LNs. **(D)** CNR of LNs stained with different concentration of I_2_-IK for different times. **(E)** H&E staining of normal LNs in the different groups.

Before imaging LNs from clinical patients, LNs from Balb/c nude mice were used to pave the way for subsequent clinical studies. After the mice were euthanized, 20 normal LNs were dissected and divided into four groups, followed by formalin fixation for 12 h. Then, the LNs were stained with different concentrations of I_2_-IK (w/v, the concentration of iodine was 0%, 0.5%, 1%, and 2%) for 24 h. During the staining, micro-CT imaging was performed at 0.5, 1, 2, 4, 8, and 24 h (**Figure 1A**). CT value in the periphery region, in the center region of LNs and CNR [contrast-to-noise ratio (CNR) = CT periphery/CT center], was adopted as the evaluation index of micro-CT image quality (**Figures 1B–D**). The CT value in the periphery region and center region of LNs was increased with the increased staining time (p < 0.01) and I_2_-IK concentration (p < 0.01). However, CNR was increased early time and decreased late in time on the whole. The highest CNR presented at 4 h for 0.5% group (2.03 ± 0.30), at 2 h for 1% group (1.80 ± 0.38), and at 0.5 h for 2% group (1.53 ± 0.15). Obviously, the higher the I_2_-IK concentration, the shorter the staining time needed for good imaging quality. It was also easy to produce overstaining with high concentration of I_2_-IK. Therefore, 0.5% I_2_-IK staining for 4 h was the optimal parameter for micro-CT imaging of normal LNs from mice. To further observe the influence of I_2_-IK staining in LNs, H&E staining was performed in all LNs after I_2_-IK enhanced-micro-CT imaging. The results showed that there was no influence in the quality of H&E sections with different concentrations of I_2_-IK staining (**Figure 1E**).

### I_2_-IK-Enhanced Micro-CT Image Could Successfully Detect Metastatic Foci in LNs From Melanoma Mice Model

**Figure 2:**
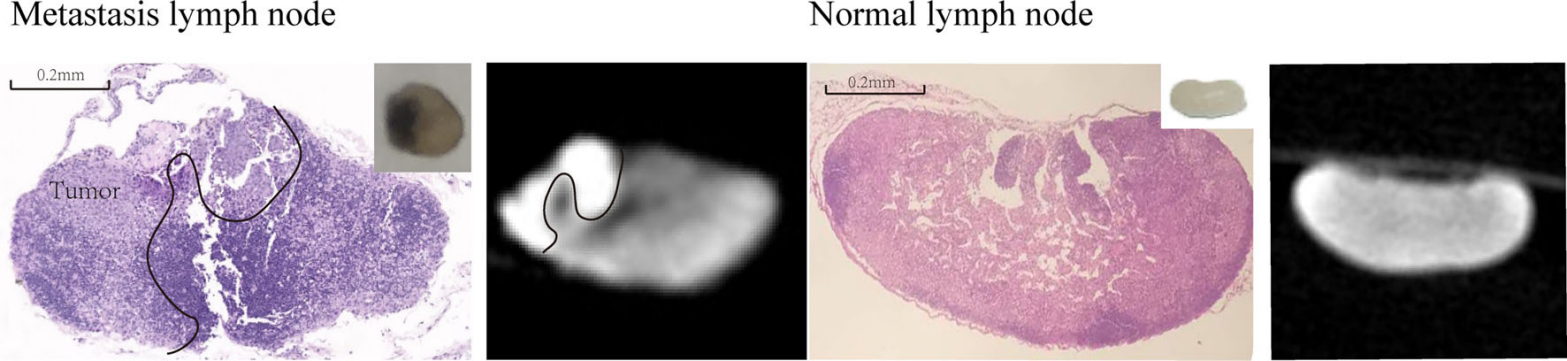
I_2_-IK-enhanced micro-CT image of melanoma metastatic LNs and normal LNs. The left figure is the H&E sections and micro-CT image of melanoma metastatic LNs, while right figure is the H&E sections and micro-CT image of normal LNs. Insert figure the camera image of melanoma metastatic LNs and normal LNs.

After the optimal parameters of I_2_-IK-enhanced micro-CT were determined, B16 melanoma LNs metastasis nude mouse model was constructed. Five out of 10 nude mice successfully developed popliteal LNs metastases. Among them, four LNs had partial metastases, and one LN had been replaced by metastasis. Micro-CT imaging was performed on all four partial metastatic LNs after being fixed in formalin for 12 h and stained with 0.5% I_2_-IK for 4 h. The results showed that the CT images of partial LNs were heterogeneous, while the CT images of normal LNs were homogeneous. The CT value of tumor tissues in LNs was significantly higher than that of normal tissues (CT_Tumor_ = 4231.5 ± 104.4, CT_normal_ = 3068.0 ± 81.3, p = 0.001) (**Figure 2**). After micro-CT imaging, these LNs underwent further H&E staining, and the results confirmed that the high intensity region in micro-CT image was metastatic tumor tissues (**Figure 2**).

### I_2_-IK-Enhanced Micro-CT Image Could Locate Metastatic Foci in LNs From OSCC Patients

**Figure 3:**
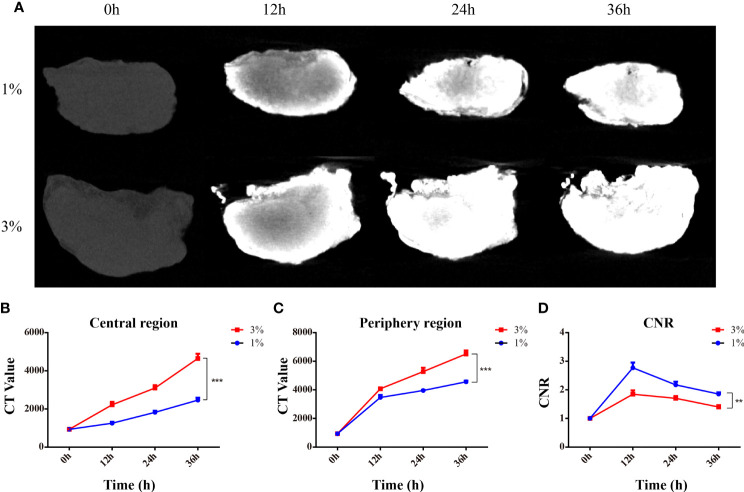
I_2_-IK-enhanced micro-CT imaging for clinical OSCC normal LNs. **(A)**, Micro-CT image of normal LNs stained with different concentration of I_2_-IK for different time. **(B, C)**, CT value in the peripheral region and central region of LNs. **(D)** CNR of LNs stained with different concentrations of I_2_-IK for different times. (**p < 0.01, ***p < 0.001).

To determine the optimal parameter for I_2_-IK-enhanced micro-CT image of OSCC clinical LNs, eight OSCC clinical normal LNs were acquired. LNs were randomly divided into two groups and fixed in formalin solution for 12 h. Then, the LNs were stained with different concentrations of I_2_-IK (w/v, the concentration of iodine was 1% and 3%) for 36 h. During the staining period, micro-CT imaging was performed per 12 h (**Figure 3A**). The evaluation index for micro-CT image quality was the same as above. With the increase in staining time (p < 0.001) and I_2_-IK concentration (p < 0.001), CT value in the periphery region and center region of LNs were both increased. However, CNR were increased early (<12 h) and decreased later (>12 h) on the whole. Among them, when 1% I_2_-IK staining for 12 h, CNR of micro-CT image reached the peak (CNR = 2.77 ± 0.18) (**Figures 3B–D** **)**.

**Figure 4:**
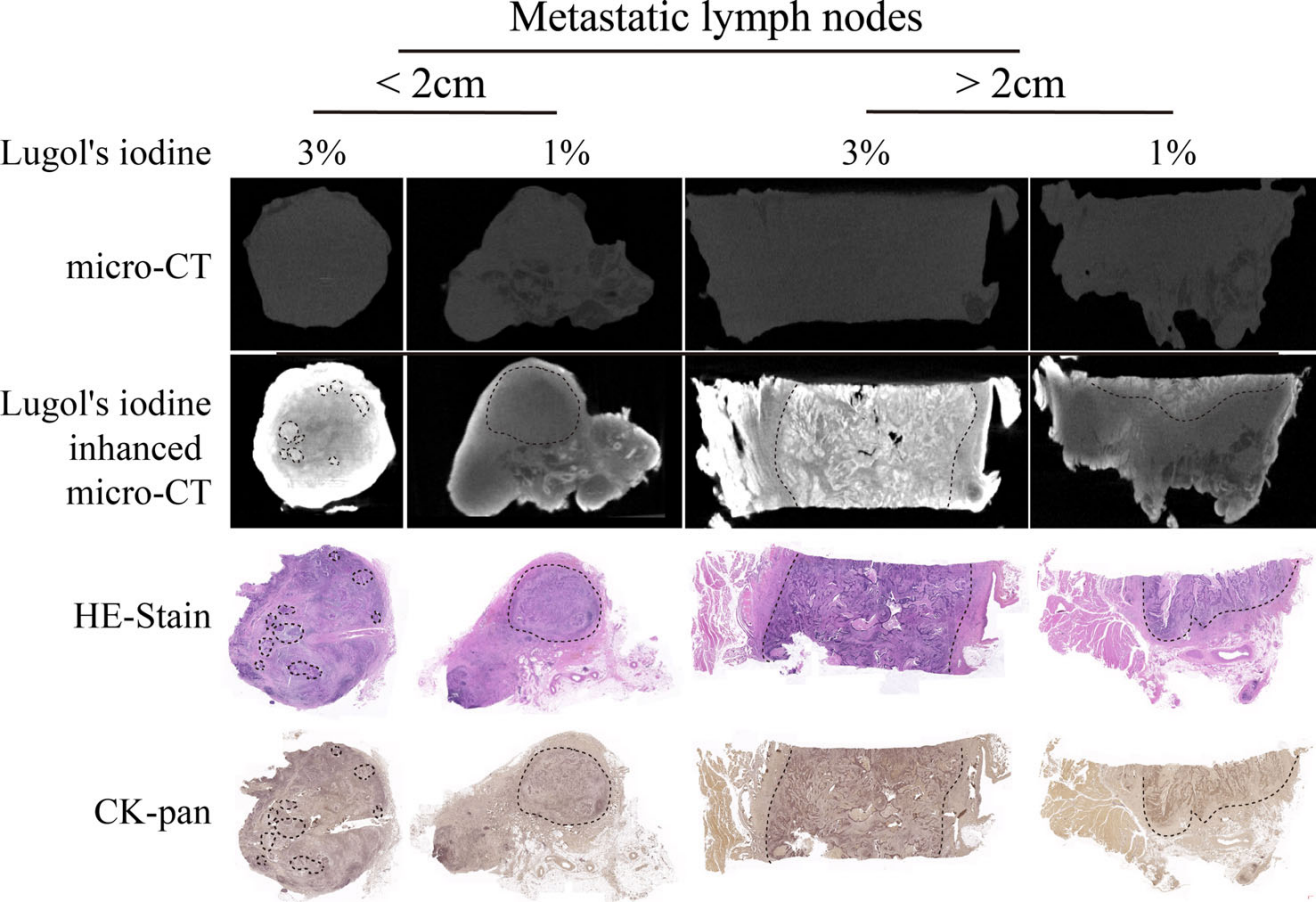
I_2_-IK-enhanced micro-CT imaging before paraffin embedding for clinical OSCC regional metastatic LNs. After comparing the image of I_2_-IK-enhanced micro-CT with simple micro-CT image, we found that I_2_-IK could enhance the contrast between metastatic tumor tissues and normal LNs tissues and 3% I_2_-IK staining acquired better effect then 1% I_2_-IK. In addition, after comparing the image of I_2_-IK-enhanced micro-CT with subsequent pathological image, the region of high CT value was highly correlated with tumor tissues.

After I_2_-IK-enhanced micro-CT imaging for normal LNs, another eight metastatic LNs were acquired to conduct I_2_-IK-enhanced micro-CT imaging. Among them, four LNs were larger than 2 cm in size, and four LNs were smaller than 2 cm in size. Given that, the volume of suspected metastatic LNs was larger than normal LNs. These LNs were also divided into 1% and 3% I_2_-IK groups (four LNs per group according to the volume). All LNs underwent micro-CT imaging before paraffin embedding. By comparing the micro-CT image with pathological image, we found that I_2_-IK-enhanced micro-CT imaging could clearly locate the metastatic foci in LNs (**Figure 4**). The region of high CT value was highly correlated with tumor tissue. Tumor background ratio (TBR = CT_tumor_/CT_normal_) was adopted to determine the optimal parameter for metastatic LNs. According to the TBR value, 3% I_2_-IK staining was more valuable in showing metastatic foci than 1% I_2_-IK staining (TBR_3%_ = 1.89 ± 0.10, TBR_1%_ = 1.28 ± 0.07, p < 0.001). However, we also found that 3% I_2_-IK staining for 12 h will led to overstaining in the periphery region of LNs and increased the fragility of nonmetastatic LNs (**Figure 4**).

**Figure 5:**
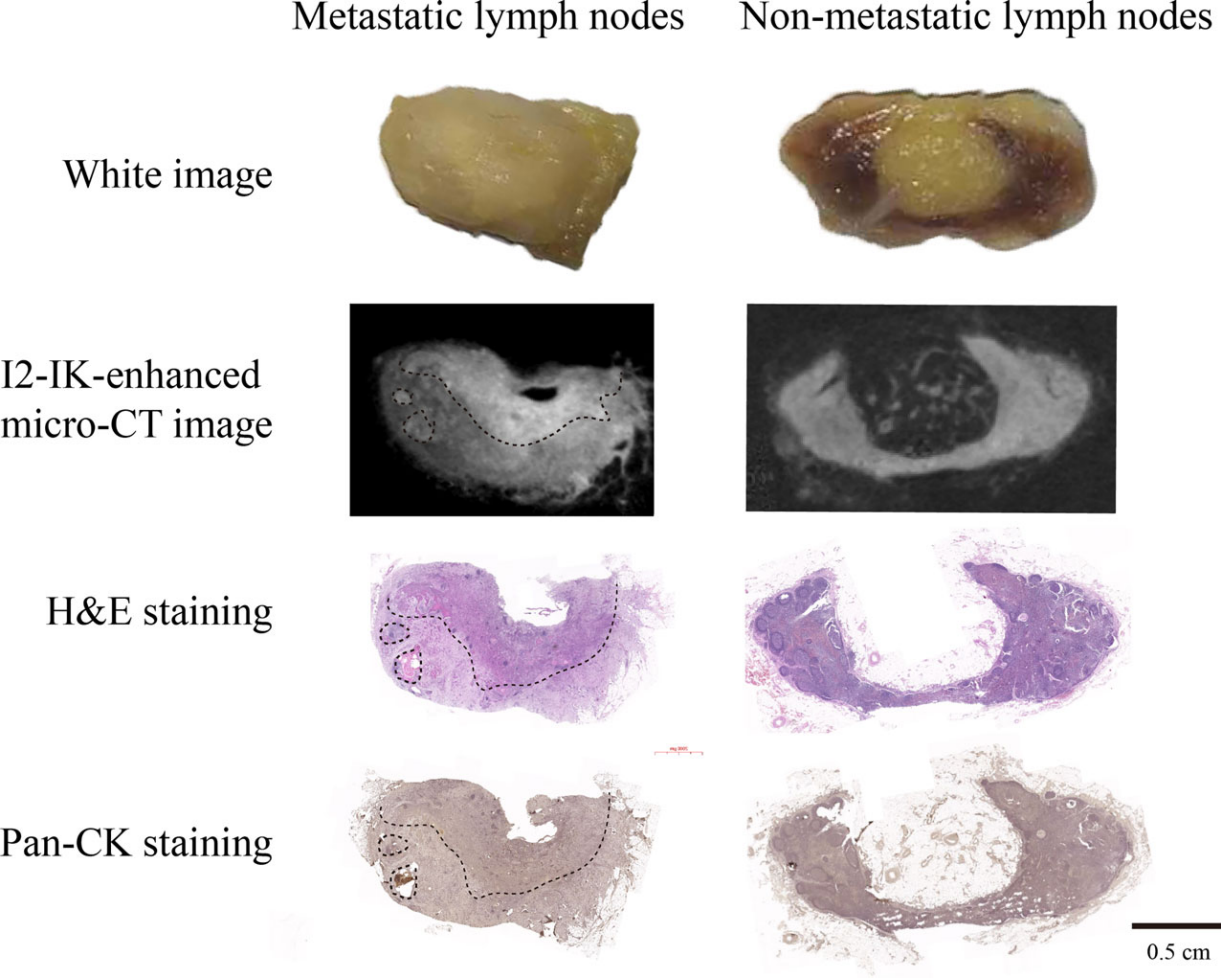
I_2_-IK-enhanced micro-CT imaging after paraffin embedding for clinical OSCC regional metastatic LNs. From the micro-CT image and pathological image, we found that I_2_-IK-enhanced micro-CT imaging could still clearly locate the metastatic foci in LNs. Moreover, the overstaining caused by high concentration I_2_-IK was disappeared after the paraffin-embedding process.

To observe the influence of paraffin-embedding process in the micro-CT image, all LNs underwent micro-CT imaging again after paraffin embedding. The results showed that after the paraffin-embedding process, I_2_-IK-enhanced micro-CT imaging could also clearly locate the metastatic foci in LNs (**Figure 5**). Moreover, the overstaining phenomenon caused by high concentration of I_2_-IK staining also disappeared (**Figure 5**).

### The Glycogen Content in Nonmetastatic LNs Tissue and Metastatic LNs Tissues

**Figure 6:**
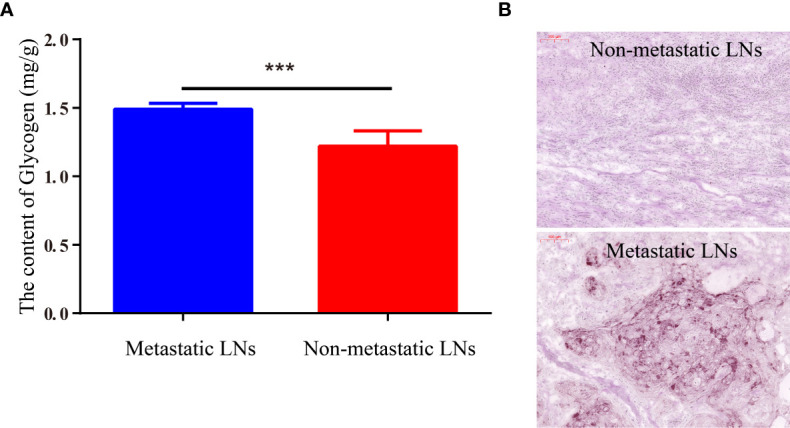
The content of glycogen in metastatic LNs and nonmetastatic LNs. **(A)** The quantitative content of glycogen detected by Liver/Muscle Glycogen Assay Kit. **(B)** PAS staining image of LNs with/without metastasis (***p < 0.001).

To further explore the explain the imaging differences between normal LN tissue and metastatic tumor tissue, the glycogen content was detected with the Liver/Muscle Glycogen assay kit. The results showed that the glycogen content of metastatic LNs tissue was significantly higher than nonmetastatic LN tissue (1.49 ± 0.04 *vs*. 1.22 ± 0.11, p < 0.001) (**Figure 6A**). For further validation of the content of glycogen in different tissues, metastatic and nonmetastatic LN tissues were also stained with PAS. The results showed that the content of glycogen in metastasis LN tissue were higher than nonmetastatic LN tissue (**Figure 6B**).

## Discussion

In the biological fields, I_2_-IK-enhanced micro-CT imaging was mainly introduced into the research of anatomical structure of various animals including invertebrates and vertebrate embryos (20, 26). The results showed that with the enhancement of I_2_-IK, micro-CT imaging could reveal with remarkable clarity which epithelial, muscular, and neural anatomy can be visualized in a range of very small specimens. In oncology, I_2_-IK-enhanced micro-CT imaging has been applied to image primary tumors such as lung cancer, and craniopharyngioma (19, 24, 27). The micro-CT image could clearly show the 3D-structure of tumor tissues, which can be highly correlated with traditional histology and immunohistochemistry. In our study, metastatic LNs were acquired to conduct I_2_-IK-enhanced micro-CT. Micro-CT image clearly showed that the intensity of tumor tissues was higher than the peripheral normal LN tissue. The area of tumor tissues was highly correlated with H&E staining and pan-CK immunohistochemical staining. Moreover, based on the micro-CT imaging of human LNs, we speculate that the micro-CT image of LN replaced by metastasis would be heterogeneous and the CT value will higher than normal LNs. Hence, there is no doubt that it is of great valuable to examine pathological samples with the assistance of I_2_-IK-enhanced micro-CT imaging.

As we all know, iodine (I_2_) could reacted with starch and glycogen and produce blue color and brown color, respectively. Previous studies (22) also indicated that the different affinity of I_2_-IK for different tissues may be related to the content of glycogen. Moreover, in the micro-CT image, the CT value of muscle around the metastatic LNs was higher than that in the LN tissues. This is in accord with the fact that muscles are rich in glycogen. In addition, in our previous study, we also found that I_2_-IK has different affinity for OSCC cells in culture with high and low glycogen (25). It reminded us that the glycogen content of tumor tissues maybe richer than that in normal tissue in LNs. In this study, we performed two different methods to detect the content of glycogen in metastatic and normal tissues in metastatic LNs. The results showed that the content of glycogen in metastatic tissue was higher than that in normal tissue in LNs. It provides the physiological basis for the application of I_2_-IK-enhanced micro-CT imaging for metastatic LNs.

The concentration of I_2_-IK and staining time were the two most important parameters for micro-CT imaging. According to the literature, staining with low concentrations of I_2_-IK needed more time for staining and had little contrast between different tissues, while staining with high concentrations of I_2_-IK may lead to overstaining, which reduces the contrast (23, 26, 28). Therefore, it is necessary to find the best concentration of Lugol’s iodine and staining time. In this study, by setting different concentrations of I_2_-IK and staining time, we found that staining with 3% I_2_-IK for 12 h was the optimal parameter for clinical OSCC metastatic LNs micro-CT imaging. However, the volume of metastasis LNs in this study was larger than that of normal LNs, and for small LNs with micro-metastasis, 1% concentration of I_2_-IK may be more superior to 3%. Moreover, the study was limited by the collection of metastasis LNs; only eight metastasis LNs were acquired for this study. There is still a need for more metastatic LNs samples to further determine the staining parameter for LNs with different volumes.

At present, the intraoperative treatment of regional LNs is blind due to the limitation of preoperative imageological examination such as CT and MRI (7, 29). Although sentinel LN biopsy (SLNB) provides the basis for the lymphadenectomy, the limitation of intraoperative pathological samples still produce a large number of false negative results (12, 30). In this study, we found that I_2_-IK-enhanced micro-CT image could clearly outline the spatial location of metastatic tissues in LNs. It could provide assistance for SLNs samples, which may decrease the false negative results of traditional pathological examination. However, in this study, the staining time of I_2_-IK staining was 12 h, which still needs to be shortened for intraoperative application.

There are still many deficiencies in this study. First, limited by the workload, serial section or semi-serial section staining could not be supplemented in this study. Second, we still cannot observe the feasibility of Lugol’s iodine enhanced micro-CT image in locating isolated tumoral cells (0.2 mm). Finally, limited by the LN samples, we have not evaluated the sensitivity and specificity of this technique. Hence, more work needs to be done to further demonstrate the value of Lugol’s iodine-enhanced micro-CT Imaging in locating micro-metastatic foci in LNs in the future.

## Conclusion

By enhanced staining with I_2_-IK, micro-CT imaging could accurately locate the 3D position of metastatic foci in regional LNs of OSCC. This method would make up the shortage of blind sampling of traditional pathological examination, which would be valuable to reduce the rate of missed metastatic LNs. In the future, more efforts still need to be made to shorten the staining time so as to improve its potential intraoperative application value.

## Data Availability Statement

The raw data supporting the conclusions of this article will be made available by the authors, without undue reservation.

## Ethics Statement

The studies involving human participants were reviewed and approved by the Institute Affiliated Stomatology Hospital, the Nanjing University Medical School. The patients/participants provided their written informed consent to participate in this study. The animal study was reviewed and approved by The Care Committee of the Nanjing University.

## Author Contributions

C-WX, S-QH, and Y-XW were responsible for the study concept and study design. C-WX, S-QH, Q-ZZ, R-LG and J-RP performed the data acquisition. C-WX, S-QH, QZ, Y-MP, and SC performed the quality control of data and algorithms, data analysis and interpretation, and statistical analysis. C-WX and S-QH prepared the manuscript. Y-XW and Q-GH edited and revised the manuscript. All authors contributed to the article and approved the submitted version.

## Funding

This study was supported by grants from Nanjing Clinical Research Center for Oral Diseases (no. 2019060009), the State Commission of Science & Technology of China (2016YFC0104100), the Jiangsu Province Science & Technology Department (BE2018618 and BE2021609), and the Nanjing Medical Science and Technique Development Foundation (QRX17174) and Clinical project, Shanghai Municipal Health Commission (202040327).

## Conflict of Interest

The authors declare that the research was conducted in the absence of any commercial or financial relationships that could be construed as a potential conflict of interest.

## Publisher’s Note

All claims expressed in this article are solely those of the authors and do not necessarily represent those of their affiliated organizations, or those of the publisher, the editors and the reviewers. Any product that may be evaluated in this article, or claim that may be made by its manufacturer, is not guaranteed or endorsed by the publisher.

## Abbreviations

3D, three-dimensional; CNR, contrast-to-noise ratio; CT, computed tomography; DAB, diaminobenzidine; H&E, hematoxylin and eosin; I_2_-IK, Lugol’s iodine; ICG, indocyanine green; IHC, immunohistochemistry; I_2_, iodine; GC, gastric carcinoma; LN, lymph nodes; OSCC, oral squamous cell carcinomas; SLNB, sentinel LNs biopsy; TBR, tumor–background ratio.

## References

[1] Siegel RL Miller KD Jemal A. Cancer Statistics, 2018. CA Cancer J Clin (2018) 68:7–30. doi: 10.3322/caac.21442 29313949

[2] Mes SW Leemans CR Brakenhoff RH. Applications of Molecular Diagnostics for Personalized Treatment of Head and Neck Cancer: State of the Art. Expert Rev Mol Diagn (2016) 16:205–21. doi: 10.1586/14737159.2016.1126512 26620464

[3] Pan J Fan Z Wang Z Dai Q Xiang Z Yuan F. CD36 Mediates Palmitate Acid-Induced Metastasis of Gastric Cancer *via* AKT/GSK-3beta/Beta-Catenin Pathway. J Exp Clin Cancer Res (2019) 38:52. doi: 10.1186/s13046-019-1049-7 30717785 PMC6360779

[4] Lee CG Jeong S Jang C Bae H Kim YH Park I. Tumor Metastasis to Lymph Nodes Requires YAP-Dependent Metabolic Adaptation. Science (2019) 363:644–9. doi: 10.1126/science.aav0173 30733421

[5] Hanai N Asakage T Kiyota N Homma A Hayashi R. Controversies in Relation to Neck Management in N0 Early Oral Tongue Cancer. Jpn J Clin Oncol (2019) 49:297–305. doi: 10.1093/jjco/hyy196 30668761

[6] Keski-Säntti H Atula T Törnwall J Koivunen P Mäkitie A. Elective Neck Treatment *Versus* Observation in Patients With T1/T2 N0 Squamous Cell Carcinoma of Oral Tongue. Oral Oncol (2006) 42:96–101. doi: 10.1016/j.oraloncology.2005.06.018 16256414

[7] Ishida T Hijioka H Kume K Yoshimura T Miyawaki A Nozoe E. A Diagnosis System for Detecting Cervical Lymph Node Metastasis in Oral Squamous Cell Carcinoma: Collective Consideration of the Results of Multiple Imaging Modalities. J Oral Maxillofac Surg Med Pathol (2017) 29:210–6. doi: 10.1016/j.ajoms.2016.12.007

[8] Chen M Chou H Liu F Chen C Lin G Yang L. ^(18)^F-FDG PET in Small-Cell Cervical Cancer: A Prospective Study With Long-Term Follow-Up. Eur J Nucl Med Mol Imaging (2016) 43:663–74. doi: 10.1007/s00259-015-3229-9 26519293

[9] Takahashi Y Suzuki S Matsutani N Kawamura M. ^18^F-Fluorodeoxyglucose Positron Emission Tomography/Computed Tomography in the Evaluation of Clinically Node-Negative Non-Small Cell Lung Cancer. Thorac Cancer (2019) 10:413–20. doi: 10.1111/1759-7714.12978 PMC6397908 30666803

[10] Wagner JM Alleman AM. Ultrasonography of Cervical Lymph Nodes. Radiol Clin North Am (2019) 57:485–500. doi: 10.1016/j.rcl.2019.01.005 30928073

[11] Onozato ML Hammond S Merren M Yagi Y. Evaluation of a Completely Automated Tissue-Sectioning Machine for Paraffin Blocks. J Clin Pathol (2013) 66:151–4. doi: 10.1136/jclinpath-2011-200205 21900334

[12] Zeng HC Hu JL Bai JW Zhang GJ. Detection of Sentinel Lymph Nodes With Near-Infrared Imaging in Malignancies. Mol Imaging Biol (2019) 21:219–27. doi: 10.1007/s11307-018-1237-4 29931432

[13] Gilmore DM Khullar OV Jaklitsch MT Chirieac LR Frangioni JV Colson YL. Identification of Metastatic Nodal Disease in a Phase 1 Dose-Escalation Trial of Intraoperative Sentinel Lymph Node Mapping in Non-Small Cell Lung Cancer Using Near-Infrared Imaging. J Thorac Cardiovasc Surg (2013) 146:562–70; discussion 569–70. doi: 10.1016/j.jtcvs.2013.04.010 PMC3748170 23790404

[14] Ferris RL Stefanika P Xi L Gooding WE Seethala RR Godfrey TE. Rapid Molecular Detection of Metastatic Head and Neck Squamous Cell Carcinoma as an Intraoperative Adjunct to Sentinel Lymph Node Biopsy. Laryngoscope (2012) 122:1020–30. doi: 10.1002/lary.22467 PMC3418874 22447185

[15] Tang R Buckley JM Fernandez L Coopey S Aftreth O Michaelson J. Micro-Computed Tomography (Micro-CT): A Novel Approach for Intraoperative Breast Cancer Specimen Imaging. Breast Cancer Res Treat (2013) 139:311–6. doi: 10.1007/s10549-013-2554-6 23670129

[16] Umetani K Okamoto T Saito K Kawata Y Niki N. 36M-Pixel Synchrotron Radiation Micro-CT for Whole Secondary Pulmonary Lobule Visualization From a Large Human Lung Specimen. Eur J Radiol Open (2020) 7:100262. doi: 10.1016/j.ejro.2020.100262 32984451 PMC7495051

[17] Troschel FM Gottumukkala RV Dicorpo D Mario J Ott HC Wright CD. Feasibility of Perioperative Micro–Computed Tomography of Human Lung Cancer Specimens: A Pilot Study. Arch Pathol Lab Med (2019) 143(3):319–25. doi: 10.5858/arpa.2018-0249-OA 30457896

[18] Tang R Saksena M Coopey SB Fernandez L Buckley JM Lei L. Intraoperative Micro-Computed Tomography (Micro-CT): A Novel Method for Determination of Primary Tumour Dimensions in Breast Cancer Specimens. Br J Radiol (2016) 89(1058):20150581. doi: 10.1259/bjr.20150581 26568439 PMC4985207

[19] Metscher BD. MicroCT for Developmental Biology: A Versatile Tool for High-Contrast 3D Imaging at Histological Resolutions. Dev Dyn (2009) 238:632–40. doi: 10.1002/dvdy.21857 19235724

[20] Metscher BD. MicroCT for Comparative Morphology: Simple Staining Methods Allow High-Contrast 3D Imaging of Diverse Non-Mineralized Animal Tissues. BMC Physiol (2009) 9:11–1. doi: 10.1186/1472-6793-9-11 PMC2717911 19545439

[21] Degenhardt K Wright AC Horng D Padmanabhan A Epstein JA. Rapid 3D Phenotyping of Cardiovascular Development in Mouse Embryos by Micro-CT With Iodine Staining. Circ Cardiovasc Imaging (2010) 3:314–22. doi: 10.1161/CIRCIMAGING.109.918482 PMC3059892 20190279

[22] Gignac PM Kley NJ. Iodine-Enhanced Micro-CT Imaging: Methodological Refinements for the Study of the Soft-Tissue Anatomy of Post-Embryonic Vertebrates. J Exp Zool (2014) 322:166–76. doi: 10.1002/jez.b.22561 24482316

[23] Gignac PM Kley NJ Clarke JA Colbert MW Morhardt AC Cerio D. Diffusible Iodine-Based Contrast-Enhanced Computed Tomography (diceCT): An Emerging Tool for Rapid, High-Resolution, 3-D Imaging of Metazoan Soft Tissues. J Anat (2016) 228:889–909. doi: 10.1111/joa.12449 26970556 PMC5341577

[24] Apps JR Hutchinson JC Arthurs OJ Virasami A Joshi A Zeller-Plumhoff B. Imaging Invasion: Micro-CT Imaging of Adamantinomatous Craniopharyngioma Highlights Cell Type Specific Spatial Relationships of Tissue Invasion. Acta Neuropathol Commun (2016) 4:57. doi: 10.1186/s40478-016-0321-8 27260197 PMC4891921

[25] Xia CW Gan RL Pan JR Hu SQ Zhou QZ Chen S. Lugol’s Iodine-Enhanced Micro-CT: A Potential 3-D Imaging Method for Detecting Tongue Squamous Cell Carcinoma Specimens in Surgery. Front Oncol (2020) 10:1867. doi: 10.3389/fonc.2020.550171 PMC7609877 33194607

[26] Maier J Sawall S Kachelries M. Assessment of Dedicated Low-Dose Cardiac Micro-CT Reconstruction Algorithms Using the Left Ventricular Volume of Small Rodents as a Performance Measure. Med Phys (2014) 41:051908. doi: 10.1118/1.4870983 24784387

[27] Haines BB Bettano KA Chenard M Sevilla RS Ware C Angagaw M. A Quantitative Volumetric Micro-Computed Tomography Method to Analyze Lung Tumors in Genetically Engineered Mouse Models. Neoplasia (2009) 11:39–47. doi: 10.1593/neo.81030 19107230 PMC2606117

[28] Pauwels E Van Loo D Cornillie P Brabant L Van Hoorebeke L. An Exploratory Study of Contrast Agents for Soft Tissue Visualization by Means of High Resolution X-Ray Computed Tomography Imaging. J Microsc (2013) 250:21–31. doi: 10.1111/jmi.12013 23432572

[29] Brennan PA Subramaniam S Tsioryannis C Green B. An Update on the Latest Evidence for Managing the Clinically Negative Neck (Cn0) in Oral Squamous Cell Carcinoma. Oral Dis (2017) 23:287–91. doi: 10.1111/odi.12528 27341071

[30] Qiu SQ Zhang GJ Jansen L de Vries J Schroder CP de Vries EGE. Evolution in Sentinel Lymph Node Biopsy in Breast Cancer. Crit Rev Oncol Hematol (2018) 123:83–94. doi: 10.1016/j.critrevonc.2017.09.010 29482783

